# Coats, Bags, and Puerperal Fever

**Published:** 1898-07-16

**Authors:** 


### Coats, Bags, and Puerperal Fever.

The *Lancet* has done well in urging modern views regarding the attainment of asepsis, in answer to a correspondent who asked when he might recommence midwifery practice after attending a case of puerperal fever. Every midwifery case, it is insisted, should be looked on as an operation in which the wound very easily becomes affected with sepsis, and when septic mischief has occurred very definite measures should be adopted to prevent its spread. A mere time limit is of no use. The clothes in which the patient was attended should be thrown away, or, if that be undesirable, they should be disinfected thoroughly by heat. The cuffs of the sleeves very easily get septic. It would certainly be well never to wear the coat again. The instruments are very important; all those of metal, such as scissors used for cutting the cord, and forceps, should be well boiled. Catheters should be burnt, and the whole midwifery bag, including the bottles, should be rendered aseptic. The lining of the bag especially, and the handle are very liable to transmit sepsis, and it is well to have a new lining.

These directions may, says the *Lancet*, appear extravagant and absurd, but it is useless to trust to the mere lapse of time. Puerperal fever is always caused by the transference of micro-organisms, and it is possible, with care, for the accoucheur to be quite sure that his hands and all the instruments he uses are quite free from germs. He may attend another confinement as soon as he is "aseptic," but that is a matter of careful endeavour, not of mere lapse of time. If, however, time is to be disregarded, we would urge the importance of carrying out the antiseptic measures exactly as they would be carried out by a careful surgeon in regard to operative work. It must be remembered that surgeons do not operate in their ordinary clothes. On the other hand, the accoucheur is often almost forced to do so, and thus it happens that the clothes become a very active agent in the diffusion of infection; and if they are not thrown away, as the *Lancet* suggests, they should at least be disinfected by steam. In fact, there can be but little doubt that it would be well for any man who does much midwifery to dress habitually in clothing which can be thoroughly disinfected. Perhaps in the future the ideal accoucheur will be recognised by his gloveless hands and by his flannels, well shrunk from frequent sterilisation, rather than by the conventional costume so many medical men affect.

Another great source of trouble is the midwifery bag. There can be no doubt that the leather bag is a mistake. No amount of changing the linings is of the slightest use if once the bag has become infected. The bag itself should be made of strong canvas or some similar material, so that the whole affair can be periodically boiled.

But if the accoucheur of the future is to be a shaven and closely-cropped priest of hygiene, clean and well purified from crown to sole, what about the priestesses? As to this, we cannot but feel ourselves in a difficulty. When women are employed as nurses—when, that is, one woman gives herself up to one case—the risk of her carrying disease about is reduced to a minimum. But when a woman becomes a midwife in the modern sense, that is when she "goes about like a doctor" from patient to patient, it must be obvious to all that she does her work under certain disabilities compared with the other sex.

The clothing of women is of a much more complex nature than that of men, and although this could undoubtedly be altered, such alteration has not as yet made much headway. Then, above all, there is the question of the hair, which is a standing difficulty. No doubt we shall be told that midwives are perfectly ready to cut their hair in response to the demand for asepticity; but that, we fear, only means that they are willing to go about in curly locks. We seriously doubt whether many women are to be found who, for the sake of their work, are willing to have their hair cut "quite short" in the sense in which that term is used by men. This side of the question hardly came up in the old days, but the insistance with which all who practice midwifery are now urged to avoid all chance of carrying disease from patient to patient must make a considerable difference in the position of men and women in regard to midwifery practice. Custom and use has much to do with our ideas of the fit and the beautiful, and we fear that custom and use threaten to be very hard upon the midwife of the future. The ideal accoucheur will probably approach more and more every year to a figure we are all familiar with upon the river or in the cricket field, clean, short cropped, and clad in spotless flannels; but the ideal midwife will, we are afraid, cut but a funny figure.

